# Rethinking the Role of Orexin in the Regulation of REM Sleep and Appetite

**DOI:** 10.3390/nu15173679

**Published:** 2023-08-22

**Authors:** Maria P. Mogavero, Justyna Godos, Giuseppe Grosso, Filippo Caraci, Raffaele Ferri

**Affiliations:** 1 Department of Psychology, Vita-Salute San Raffaele University, 20132 Milan, Italy; paola_mogavero@libero.it; 2 San Raffaele Scientific Institute, Division of Neuroscience, Sleep Disorders Center, 20127 Milan, Italy; 3 Department of Biomedical and Biotechnological Sciences, University of Catania, 95123 Catania, Italy; justyna.godos@unict.it (J.G.); giuseppe.grosso@unict.it (G.G.); 4 Neuropharmacology and Translational Neurosciences Research Unit, Oasi Research Institute—IRCCS, 94018 Troina, Italy; fcaraci@unict.it; 5 Department of Drug and Health Sciences, University of Catania, 95125 Catania, Italy; 6 Sleep Research Centre, Oasi Research Institute—IRCCS, 94018 Troina, Italy

**Keywords:** orexin, hypocretin, REM sleep, feeding, appetite, sleep, wakefulness, eating disorders

## Abstract

Orexin plays a significant role in the modulation of REM sleep, as well as in the regulation of appetite and feeding. This review explores, first, the current evidence on the role of orexin in the modulation of sleep and wakefulness and highlights that orexin should be considered essentially as a neurotransmitter inhibiting REM sleep and, to a much lesser extent, a wake promoting agent. Subsequently, the relationship between orexin, REM sleep, and appetite regulation is examined in detail, shedding light on their interconnected nature in both physiological conditions and diseases (such as narcolepsy, sleep-related eating disorder, idiopathic hypersomnia, and night eating syndrome). Understanding the intricate relationship between orexin, REM sleep, and appetite regulation is vital for unraveling the complex mechanisms underlying sleep-wake patterns and metabolic control. Further research in this field is encouraged in order to pave the way for novel therapeutic approaches to sleep disorders and metabolic conditions associated with orexin dysregulation.

## 1. Introduction

Sleep is a complex physiological process that consists of different stages, including rapid eye movement (REM) sleep. REM sleep is characterized by vivid dreaming, rapid eye movements, and muscle atonia. Although not completely understood, it is clear that REM sleep accomplishes many fundamental functions: information processing by replaying neural activity experienced during wakefulness, modulation of synaptic and neural transmission, cortical plasticity, memory consolidation, and a series of physical functions particularly interesting for this review, such as the regulation of feeding behavior, autonomic nervous system, body temperature, and response to stress, among many others [1].

The regulation of REM sleep is a finely tuned process involving various neurotransmitters and neuropeptides [2], among them orexin plays a pivotal role. The regulation of the sleep-wake cycle and of the various stages of sleep is very complex and still under study, according to current knowledge, among the neurons involved in the regulation of REM sleep there are the cholinergic ones, which are activated in association with fast cortical rhythms also during wakefulness, but much less during NREM sleep [3]. Much less is known about glutamatergic neurons, although it is known that, by innervating the cortex and subcortical regions that promote arousal, they appear to fire during wakefulness and REM sleep. Furthermore, the GABAergic neurons in the Pedunculopontine and Laterodorsal Tegmental Nuclei regions seem mainly wake-active, but some may be more active during REM sleep than in NREM sleep [3]. Orexin-A and -B are neuropeptides essential for regulating wake and REM sleep [1]; indeed, intracerebroventricular injection of orexin-A increases wake and suppresses REM sleep for several hours and chemogenetic activation of the orexin neurons increases wake and strongly suppresses REM sleep [4]. The orexin neurons also produce glutamate and the inhibitory neuropeptide dynorphin, and co-release of glutamate from the orexin neurons can excite target neurons [5], suggesting a close interconnection with the aforementioned neurotransmitter pathways, within the control of the REM sleep, although further studies are needed.

Since its identification, in 1998, orexin has been known to regulate feeding behavior [6]. This also determined the choice of its name from the Ancient Greek word ὄρεξις (órexis, meaning “desire” or “appetite”). An independent group, at the same time, identified it as “hypocretin” [7] and underlined its similarity with the gut hormone secretin. For this reason, and due to its hypothalamic origin, these authors choose the name “hypocretin”. Soon after, orexin and hypocretin were recognized to be the same neurotransmitter and there is now a general agreement on the use of the term hypocretin to refer to the gene or genetic products and orexin to indicate the protein.

It should be said that orexin includes two similar peptides, deriving from the hypothalamic prepro-orexin, called orexin A and B, corresponding to hypocretin 1 and 2, and formed by 33 and 28 amino acids, respectively. Also, two different orexin receptors have been identified (orexin receptor 1 and 2); orexin A acts through both receptor 1 and 2 while orexin B only acts through receptor 2 [8]. However, in this article we will not distinguish between these two peptides, in order to keep the text more easily readable.

As seen above, orexin plays a significant role in the modulation of REM sleep, as well as in the regulation of appetite [9]. This article explores, briefly, the relationship between orexin, REM sleep, and appetite regulation, with the aim to provide a reappraisal of the role of orexin in the regulation of REM sleep and to shed light on the interconnected nature between them and appetite.

## 2. The Role of Orexin in REM Sleep Regulation

Orexin is primarily synthesized in the lateral hypothalamus and acts as a neurotransmitter. According to the current knowledge, it plays a key role in the regulation of sleep-wake cycles, promoting wakefulness and inhibiting REM sleep. Orexin-producing neurons in the hypothalamus project to several brain regions involved in sleep regulation, such as the brainstem and thalamus [10].

During wakefulness, orexin neurons are highly active, releasing orexin into their target regions. The release of orexin promotes arousal, enhances alertness, and helps maintain a state of wakefulness. However, during REM sleep, the activity of orexin neurons decreases, resulting in a reduction of orexin release. Interestingly, recent studies in primates have clearly shown that the highest concentrations of orexin are recorded until just after the monkeys go to sleep and then falling through the night and reaching the lowest concentrations around wake time [11,12]. Then, orexin concentrations start rise linearly. Only after some hours of wakefulness, orexin concentrations reach a plateau during the early evening. Data in humans, although less consistently, agree with this circadian distribution of orexin levels in the cerebrospinal fluid (CSF) [13,14,15].

This pattern is definitely not what one should expect if one of the main roles of orexin is that of maintaining wakefulness, since it would be quite difficult to explain why orexin is at its lowest levels in the morning hours when alertness is maximum and sleepiness is unlikely and is maximum around the sleep onset, when sleepiness is likely to be maximum. On the contrary, it fits quit well with the notion that inhibits REM sleep and, indeed, it decreases gradually during the night, concurrently with the well know increase in REM sleep as the night progresses.

The discrepancy between the supposed alerting role of orexin and the CSF data has also been supposed to be due to a hypothetical long delay between the release of orexin and its appearance in lumbar CSF [15]. However, this was only a suggestion based on no evidence and a much easier interpretation of the data is that the main role of orexin is not that of promoting alertness.

**Figure 1:**
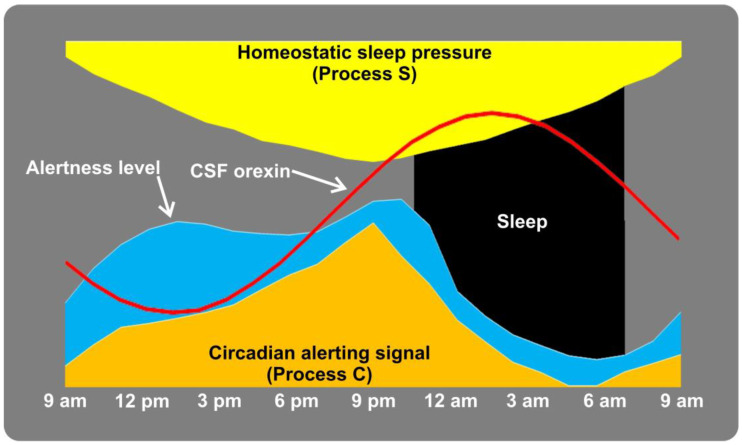
Schematic representation of the complex interplay between sleep pressure (Process S), alerting signals (Process C) [14,16], alertness level and CSF orexin levels [15] along the 24-h sleep/wake cycle.

Figure 1 shows, schematically, the complex interplay between sleep pressure (Process S), alerting signals (Process C), alertness level and CSF orexin levels along the 24-h sleep/wake cycle [14,16].

Considering orexin essentially as a neurotransmitter inhibiting REM sleep and, to a much lesser extent, a wake promoting agent, also fits with the objective observations reported about sleep in patients with narcolepsy type 1 in whom a deficit in orexin represents the main biological hallmark. In fact, these patients show full (sleep onset REM sleep episodes) or partial (sleep paralysis, cataplexy, hypnagogic hallucinations) intrusions of REM sleep during wakefulness. In addition, nocturnal sleep is also disturbed in these patients and is not characterized by an increased nonREM sleep time but, again, by an increase in REM sleep which also occurs early after thee subjects fall asleep.

In physiological conditions, the decrease in orexin activity during the second part of the night, when REM sleep preferentially occurs, is essential for the initiation and maintenance of this sleep stage. By inhibiting orexin release, the brain allows for the expression of REM-specific features, such as muscle atonia and vivid dreaming.

There is a general idea that one of the main roles of orexin is to promote wakefulness [17]. This has also represented at least part of the rationale to promote orexin receptor antagonists for the treatment of insomnia and to promote sleep [18]. However, although several neurotransmitters, including hypocretin/orexin, histamine, norepinephrine, serotonin, dopamine, adenosine and acetylcholine, certainly contribute to the mechanisms supporting wakefulness, none of them seem to be individually necessary for maintaining wakefulness [19].

Orexin receptor antagonists are a class of medications designed to block the action of orexin at its receptors. By doing so, these drugs are believed to inhibit the wake-promoting effects of orexin, ultimately leading to increased sleep duration and improved sleep continuity. Numerous polysomnographic studies have investigated the effects of orexin receptor antagonists on sleep architecture in both healthy individuals and patients with sleep disorders [18]. These studies have consistently shown that orexin receptor antagonists improve sleep parameters and promote sleep continuity. Studies have generally reported that the administration of orexin receptor antagonists was associated with a significant increase in total sleep time and reduced sleep latency, but also enhanced or at least preserved REM sleep, unlike traditional hypnotics, and sometimes decreased nonREM sleep [20]. It is intuitive to think that if orexin essentially favors wakefulness, antagonizing its receptors should enhance sleep as a whole, especially nonREM sleep which is physiologically more represented than REM sleep. On the contrary, orexin receptor antagonists have shown a remarkable ability to enhance REM sleep regulation, in particular [20].

For all the above reasons, it is our opinion that a careful rethinking and reconsideration of the main role of orexin should be carried out in the scientific literature since this would translate into a different pharmacological approach to the treatment of conditions in which orexin is deficient (narcolepsy type 1) [21] or is believed to be upregulated, such as insomnia [18].

## 3. Other Functions of Orexin

Orexin, however, plays a multifunctional role in regulating various physiological processes. Besides sleep-wake regulation and appetite control, its functions encompass energy homeostasis, autonomic nervous system modulation, and cognitive function, reflecting its significance in maintaining overall homeostasis.

### 3.1. Energy Homeostasis

Beyond its involvement in feeding behavior, orexin is crucial in maintaining energy homeostasis. It orchestrates energy expenditure, thermogenesis, and lipid metabolism through interactions with various metabolic centers in the brain. Studies in animal models suggest that orexin deficiency can lead to reduced physical activity and increased fat accumulation, underscoring its role in energy balance [22,23].

Energy homeostasis is a crucial physiological process that maintains a balance between energy intake and energy expenditure in the body. Dysregulation of this process can lead to obesity, diabetes, and other metabolic disorders. Orexin-producing neurons in the lateral hypothalamus are activated by both fasting and food deprivation, suggesting a role in promoting food-seeking behavior [6]. These neurons project widely to other brain regions involved in appetite regulation, such as the paraventricular thalamic nucleus and the nucleus accumbens [24]. Activation of orexin neurons stimulates food intake, while inhibition reduces food consumption [25].

Beyond its role in feeding behavior, orexin also influences energy expenditure. Orexin receptors are expressed in various tissues, including brown adipose tissue and skeletal muscles, which are involved in thermogenesis and energy dissipation [26,27].

Orexin also has an impact on glucose homeostasis. It interacts with insulin-producing beta cells in the pancreas and affects glucose-stimulated insulin secretion [28,29].

Leptin, a hormone produced by adipose tissue, is a key player in energy homeostasis by regulating satiety and energy expenditure. Orexin and leptin interact in a complex manner to regulate feeding behavior and energy balance [30].

Thus, orexin is a neuropeptide that plays a significant role in the regulation of energy homeostasis by influencing feeding behavior, energy expenditure, and glucose homeostasis. Its complex interactions with other hormones, such as leptin, further highlight its importance in maintaining energy balance. Dysregulation of orexin signaling has been implicated in metabolic disorders, making it a potential pharmacological target for therapeutic interventions aimed at addressing obesity and related conditions.

### 3.2. Regulation of the Autonomic Nervous System

Orexinergic pathways also play a role in the stress response, contributing to the activation of the hypothalamic-pituitary-adrenal axis. Dysregulation of orexin signaling is associated with autonomic dysfunction and cardiovascular disorders [31,32,33].

The autonomic nervous system is a critical component of the peripheral nervous system responsible for regulating involuntary physiological functions, such as heart rate, blood pressure, digestion, and respiratory rate. Emerging evidence suggests that orexin also plays a significant role in modulating the autonomic nervous system and it has been shown to influence the activity of both the sympathetic [34] and parasympathetic [35] nervous systems [36]. Moreover, orexin is involved in the regulation of the cardiovascular [34] and respiratory [37] functions through its actions on the autonomic nervous system. Dysregulation of orexin signaling may contribute to autonomic dysfunctions, making it a potential target also for therapeutic interventions in disorders involving autonomic nervous system imbalances.

### 3.3. Cognitive Function

Cognitive function refers to the mental processes that enable us to perceive, think, reason, and remember. Emerging evidence suggests that orexin may also influence cognitive function, including memory consolidation and attention. Orexin receptors are found in brain areas related to memory, and studies have linked orexin to the modulation of cognitive processes. Moreover, orexin has been implicated in the regulation of emotional responses and mood, further supporting its involvement in cognitive function [38,39].

Arousal is a critical component of cognitive function, as it affects attention, alertness, and information processing. As also reported above, orexin has a significant influence on arousal and wakefulness [40], as well as on attention (the ability to focus on specific stimuli while filtering out irrelevant information) [41]. Learning and memory are fundamental cognitive functions essential for acquiring and retaining new information; orexin may influence these processes through its enhancing effects on synaptic plasticity [42]. However, the exact mechanisms of orexin’s impact on cognitive function certainly require further investigation, also for their potential implications for developing novel therapeutic strategies targeting cognitive dysfunction in CNS disorders.

## 4. The Relationship between Orexin, REM Sleep, and Appetite

In addition to its role in sleep regulation, orexin is also involved in the regulation of appetite and energy homeostasis. Orexin neurons project to the lateral hypothalamus, a region known to be involved in appetite control. Through its interaction with other brain regions, orexin helps regulate feeding behavior and energy balance [43].

In physiological conditions, REM sleep is most abundant towards the end of the main nocturnal sleep episode. As nocturnal sleep lasts for hours, it is accompanied by a physiological fasting, and the abundant REM sleep in the last part of the night also acts as an appetite suppressant [44]. Consequently, REM sleep is believed to have some sort of anti-obesity function [45] and that the loss of REM sleep, especially in the last sleep hours in habitually short sleepers, might enhance appetite and contribute to weight gain. Foods are also selected for their hedonic (emotional) properties, it is reasonable to believe that REM sleep might play a role in the development of food preferences and dislikes [44].

The link between orexin, sleep, and appetite is further supported by different studies showing that disruptions in the orexin system can significantly affect food intake and metabolism [9]. Orexin-deficient animals and individuals with narcolepsy often exhibit increased food consumption, especially for high-calorie foods, indeed narcolepsy is often accompanied by weight gain and metabolic disturbances [11]. This suggests that orexin is involved in regulating food reward and the hedonic aspects of eating [46]; however, how orexin deficiency/lack translates into weight gain and metabolic disturbances is unclear and in apparent contradiction with the orexin’s facilitation of food intake and food-seeking behavior. In this respect, it should be noted that narcoleptic patients and orexin-deficient animals are hypophagic [47,48] and show body mass index-independent metabolic alterations. Thus, a complex, yet not completely known mechanism needs to be invoked to explain this contradiction.

In addition, sleep-related eating disorder (SRED), a nonREM sleep parasomnia characterized by an interruption of overnight fasting with episodes of sleep feeding [49], is very frequent in narcolepsy type 1 (in which there is orexin deficiency), but not in idiopathic hypersomnia (in CSF orexin levels are within the normal limits) [50]. Orexin receptor agonists have shown efficacy in treating narcolepsy [21]. Thus, these agents may offer a targeted approach to manage SRED by modulating orexin signaling and disrupting the abnormal sleep-related feeding patterns. However, further research is necessary to elucidate the specific molecular mechanisms underlying orexin’s involvement in SRED and to conduct clinical trials to assess the safety and effectiveness of orexin-targeted therapies in this unique sleep disorder.

Another evidence that the circadian trend of orexin can influence appetite is represented by the video-polysomnographic observation of the episodes of nocturnal feeding in patients with night eating syndrome, a condition different from the above-mentioned sleep-related eating disorder, in whom evening hyperphagia occurs with consumption of at least 25% of the daily caloric intake after the evening meal. In these patients, the duration of feeding episodes and the latency of falling asleep in the early evening hours, when orexin levels are relatively low (Figure 1) are significantly longer than in episodes of food ingestion during nocturnal awakenings in nonREM sleep, when orexin levels are high [51]. Moreover, awakenings from night sleep with feeding occur almost entirely during nonREM sleep (during which orexin is relatively high), as opposed to REM sleep (when orexin levels are lower) [51].

Furthermore, orexin interacts with other neuropeptides and neurotransmitter systems involved in appetite regulation, such as neuropeptide Y and melanin-concentrating hormone, through an involvement of the paraventricular nucleus of the hypothalamus and the nucleus of the solitary tract; these interactions contribute to the complex regulation of feeding behavior and energy balance [52].

The involvement of orexin in both sleep homeostasis and appetite could have important implications in the prevention of neurodegenerative and tumor diseases, in light of the evidence underlining a role of this neurotransmitter in both processes, according to a mechanism of inverse comorbidity [53]. After all, recent studies suggest the use of drugs that act on orexinergic receptors also in the treatment of different cancers including colon, pancreas and prostate cancers [54] and neurodegenerative diseases [55,56] such as Alzheimer’s disease (AD) where orexinergic system dysregulation promotes sleep-wake cycle impairment [57] and also interacts with CSF AD biomarkers, such as beta-amyloid and tau proteins. Along this line, the role of nutrition in sleep medicine [58] and secondary prevention strategies is continuously increasing both in oncology and neurodegeneration [59,60].

AD, for instance, is associated with early alterations in the orexinergic system. Liguori et al. [57] found significantly dysregulated orexin in the cerebrospinal fluid of AD patients, correlated to a dysregulation of their sleep-wake cycle, suggesting an impaired orexinergic tone in the disease. Similarly, Parkinson’s disease (PD) has been linked to alterations in the orexin system. A significant loss of orexin-producing neurons in the hypothalamus of PD patients, which was associated with sleep disturbances and weight loss commonly observed in PD has been reported [61].

Beyond its role in feeding behavior, emerging evidence suggests that orexin may have neuroprotective properties. Studies in animal models have demonstrated that orexin can protect against neurodegeneration by promoting cell survival and reducing oxidative stress [62]. It has also been observed that orexin seems to be implicated in neurodegeneration, with an involvement of mitochondrial processes, in both AD [63] and PD, in the latter case likely mediated by long non-coding RNAs [64] which play a role in reverse comorbidity mechanisms between cancer and neurodegeneration [65]. These findings raise the intriguing possibility that the orexin system could be a potential pharmacological target for therapeutic interventions in neurodegenerative diseases as well.

Feeding disturbances in neurodegenerative diseases are prevalent and significantly impact the overall health and quality of life of affected individuals. These disturbances can manifest as changes in appetite, alterations in eating patterns, and difficulties with swallowing or chewing. In diseases such as AD, PD, and Huntington’s disease, feeding disturbances often occur as the disease progresses and can be associated with cognitive and motor impairments [66]. For instance, individuals with AD may experience a loss of appetite and forget to eat, leading to unintended weight loss. In PD, difficulties with swallowing, known as dysphagia, can increase the risk of choking and aspiration pneumonia [67]. Understanding and managing these feeding disturbances are essential to ensure adequate nutrition, prevent complications, and enhance the overall well-being of patients with neurodegenerative diseases.

The intricate relationship between orexin and feeding behavior in neurodegenerative diseases provides a fascinating area of research with significant clinical implications. While disturbances in the orexin system have been observed in various neurodegenerative disorders, further studies are needed to fully understand the underlying mechanisms and to develop targeted therapeutic approaches. The investigation of orexin in the context of neurodegenerative diseases may lead to innovative treatment strategies and contribute to the overall understanding of the complex pathophysiology of these devastating disorders [62,68].

Moreover, in many types of cancer, an upregulation of the orexinergic system has been reported [53] that seems to have a dual effect [69]. In fact, in some tumors apoptosis has been observed associated with orexin upregulation while, in others, a proliferative effect has been found. These effects are mediated by complex metabolic changes including, among others, glucose transport and uptake and insulin. Feeding behavior and metabolism play a significant role in cancer development and progression. Obesity, which often results from excessive calorie intake and sedentary lifestyle, has been associated with an increased risk of various cancers, including glioma, colorectal, lung, and breast cancers, cervical and ovarian cancers, and cancer of the corpus uteri [70]. Orexin’s role in regulating feeding behavior and energy homeostasis implicates it as a potential mediator of the obesity-cancer link. For this reason, both antagonists or agonists of the orexin receptors are potential treatments for tumors expressing orexin receptors.

Dysregulation of the orexin system has also been implicated in the pathophysiology of obstructive sleep apnea [71]. Orexin receptor agonists have shown potential in animal studies by increasing reducing the frequency of sleep apnea events [72]. However, preliminary human studies are still contradictory [73,74]. Additionally, orexin receptor antagonists have demonstrated effectiveness in promoting sleep and decreasing wakefulness, which may be beneficial for certain types of sleep apnea [18]. However, more research is needed to fully understand the complex interactions between orexin signaling and sleep apnea and to develop safe and effective orexin-based therapies for individuals suffering from this prevalent sleep disorder. Further clinical trials evaluating the impact of orexin modulation in sleep apnea are necessary to validate its therapeutic potential and address the unmet needs in sleep apnea management.

A novel interesting research area could also evaluate the role of orexin in modulating drugs for the treatment of eating disorders (as evidenced by recent studies on animal models [75,76]), especially if also associated with sleep disorders. However, the effect of this neurotransmitter on REM sleep, which plays an important role in sleep physiology, appetite, cognitive functions and metabolism [77], should be reconsidered.

## 5. Treatment Perspectives

As our understanding of orexin’s role in the brain deepens, novel treatments that target orexin system to address several medical conditions effectively are becoming clearer. Besides the obvious possible use of orexin receptor agonists in narcolepsy and antagonists in insomnia, several other disorders might benefit from a specific action on orexin by pharmacological agents [78].

As see above, the orexin system is also involved in regulating appetite and feeding behavior, orexin receptor antagonists have been studied as potential treatments for obesity by reducing food intake and promoting weight loss [79]. However, the challenge lies in achieving a balance between appetite suppression and potential side effects. Future treatments may involve more selective orexin-targeting agents or combination therapies with other appetite-regulating systems to address obesity effectively.

In the light of what has been discussed, it is important to focus attention on the future perspectives concerning the modulation of orexinergic circuits in the treatment of metabolic disorders. In fact, orexin plays a role not only at a central level, but also at a peripheral level (gastrointestinal tract, adrenal cortex, gonads, and pancreas). Thus, the orexin peptide/receptor system is also involved in cardiovascular modulation, and neuroendocrine and reproduction regulation [80]. On the other hand, some reports have highlighted the role of orexin in the metabolic syndrome in patients with schizophrenia [81,82] and in adolescents without psychiatric pathologies [83], although further studies in this regard are also desirable in other clinical conditions.

The intertwining of the orexin system with the brain’s reward circuitry has prompted studies on its involvement in addiction and substance abuse. Orexin neurons have been found to modulate the dopaminergic system and therefore reinforcing effects of drugs of abuse, making them potential targets for addiction treatment. Animal and human studies using orexin receptor antagonists have shown promise in reducing drug-seeking behavior [84]. Future clinical trials may explore the use of orexin-targeted therapies as adjunct treatments for addiction to complement existing behavioral and pharmacological interventions.

Moreover, orexin has been implicated in anxiety and mood disorders due to its influence on stress responses, emotional regulation and affective disorders. Some studies suggest that dysregulation of the orexin system may contribute to the development of anxiety disorders, such as generalized anxiety disorder and post-traumatic stress disorder. Future treatments targeting orexin system could potentially lead to novel therapeutic options for individuals with these conditions [85,86].

Gene therapy and cell-based therapies represent cutting-edge approaches that hold significant promise for the treatment of disorders related to orexin deficiency. These techniques involve introducing genes or cells that can produce functional orexin or repair damaged orexin neurons [87,88]. For narcolepsy and other disorders characterized by orexin neuron loss, gene therapy and stem cell transplantation could potentially rescue normal orexin signaling and alleviate symptoms.

Another emerging approach in treating sleep disorders and related conditions involves using neurostimulation to modulate the activity of orexin-producing neurons. Deep brain stimulation and transcranial magnetic stimulation are examples of non-invasive or minimally invasive techniques that could be explored to regulate the activity of orexin neurons and restore balance in sleep-wake cycles [89,90].

The orexin system is a fascinating pharmacological target for future treatments of various medical conditions, particularly sleep disorders. As researchers delve deeper into the intricacies of orexin’s role in the brain and its impact on sleep regulation, appetite, addiction, mood, and more, novel therapeutic approaches are likely to emerge. From selective orexin receptor modulators to gene therapy and neurostimulation, the future holds exciting possibilities for leveraging orexin-targeted treatments to improve the lives of individuals affected by these conditions. As with any emerging medical interventions, safety and efficacy will remain primary concerns, and extensive research and clinical trials will be necessary to validate these promising approaches fully. Nevertheless, the potential for orexin-targeted therapies to revolutionize sleep medicine and other fields of neuroscience is undeniably exciting.

## 6. Conclusions

Orexin, a neuropeptide primarily produced in the lateral hypothalamus, plays a crucial role in the regulation of REM sleep and appetite. By inhibiting REM sleep and, to a much less extent, promoting wakefulness, orexin helps maintain a balance between sleep and wakefulness.

Understanding the intricate relationship between orexin, REM sleep, and appetite regulation is essential for unraveling the complex mechanisms underlying sleep-wake patterns and metabolic control. Further research in this field may pave the way to identify new pharmacological targets and to develop novel therapeutic approaches to sleep disorders and metabolic conditions associated with orexin dysregulation. Some examples are provided in the research agenda below:

- In consideration of the multiple functions of orexinergic circuits in sleep and metabolic disorders, it should be assessed the possible use of orexinergic receptor antagonists in other pathologies, in addition to the current indications;
- If orexin receptor antagonists could be a viable option for the treatment of eating disorders and metabolic disorders should be addressed with specific studies;
- Considering the interactions found with dopaminergic circuits, further studies should be carried out in humans to test the possible use of drugs acting on orexinergic circuits for the treatment of anxiety, post-traumatic stress disorder and addiction (also often associated with eating disorders);
- Considering the reports on the association between nocturnal sleep eating disorders and REM and NREM sleep, probably highly related to the modulation of orexinergic circuits, further studies on humans are desirable to better understand the pathogenetic and therapeutic role of orexin in these disorders;
- More attention should be paid to the effect of the use of orexinergic antagonists in insomnia on the appetite of these patients.
